# Development and Validation of a Revised Multidimensional Digital Health Literacy Scale: Secondary Analysis Using Cross-Sectional Data From the 2022 GetCheckedOnline Community Survey In British Columbia, Canada

**DOI:** 10.2196/78008

**Published:** 2025-12-15

**Authors:** Ihoghosa Iyamu, Pierce Gorun, Hsiu-Ju Chang, Rodrigo Sierra-Rosales, Devon Haag, Heather Pedersen, Sofia Bartlett, Nathan Lachowsky, Geoffrey McKee, Catherine Worthington, Troy Grennan, Lorie Donelle, Daniel Grace, Mark Gilbert

**Affiliations:** 1 School of Population and Public Health University of British Columbia Vancouver Canada; 2 BC Centre for Disease Control Vancouver Canada; 3 School of Public Health and Social Policy University of Victoria Victoria, BC Canada; 4 Division of Infectious Diseases Faculty of Medicine University of British Columbia Vancouver, BC Canada; 5 College of Nursing University of South Carolina Columbia United States; 6 Dalla Lana School of Public Health University of Toronto Toronto Canada

**Keywords:** digital health literacy, eHEALS, confirmatory factor analysis, psychometrics, public health, digital health

## Abstract

**Background:**

Digital technologies are reshaping health care, making digital health literacy (DHL) a critical competency for navigating online health information. Although widely conceived and measured as a unidimensional measure of DHL, the literature increasingly supports a multidimensional framing of the eHealth Literacy Scale (eHEALS). Studies propose alternative factor structures that can better inform population-level interventions, but these studies have not accounted for the ordinal nature of eHEALS response data.

**Objective:**

This study aimed to identify and validate an alternate multidimensional structure of eHEALS accounting for its ordinal response scale.

**Methods:**

Data were drawn from the 2022 GetCheckedOnline community survey of consenting English-speaking British Columbia residents aged ≥16 years who reported sexual activity in the past 12 months. Participants were recruited through geo-targeted digital advertisements, community outreach, and in-person recruitment at public events, and community locations. DHL was measured using eHEALS, with responses collected on a 5-point Likert scale. Descriptive statistics summarized eHEALS responses using means, medians, and IQRs. Exploratory and confirmatory factor analyses were used to assess the scale’s structure using polychoric correlations and standard model fit indices. Reliability and validity were evaluated using polychoric ordinal alpha, average variance extracted, and composite reliability, with missing data addressed via multiple imputation.

**Results:**

Overall, 1657 participants met inclusion criteria with a mean age of 33.0 (SD 11.77, 95% CI 32.4-33.6) years. Among these 47.3% (95% CI 44.9%-49.7%) identified as women, 30.4% (95% CI 28.1%-32.6%) identified as racialized minorities, 80.5% (95% CI 78.5%–82.3%) reported easy internet access, and 32.2% (95% CI 30.0%-34.5%) had a bachelor’s degree or higher. Across eHEALS items, median scores were 4.0 (IQR 1.0-2.0) with excellent internal consistency (polychoric ordinal α=.92). Exploratory factor analysis supported a 3-factor solution explaining 65.7% of the variance, demonstrated through confirmatory factor analysis (*χ*²_17_=71.7, *P*<.001, root-mean-square error of approximation=0.059, standardized root-mean-square residual=0.026, comparative fit index=0.969, Tucker-Lewis Index=0.948). The final model included Information Navigation (standardized loadings=0.765-0.917), Resource Appraisal (0.825-0.892), and Confidence in Use (0.803 for both items), with composite reliability (0.784-0.900), and average variance extracted (0.503-0.738) supporting construct validity.

**Conclusions:**

This study confirms a multidimensional structure of eHEALS, identifying Information Navigation, Resource Appraisal, and Confidence in Use as key dimensions of DHL. This revised model enhances measurement precision, enabling more accurate identification of populations with limited DHL and informing the development of targeted, equity-oriented interventions. Future research should aim to confirm this multidimensional structure in more diverse populations and explore how distinct DHL domains influence access to digital health services in various contexts. Additionally, ongoing scale development must adapt to account for the role of emerging technologies, including artificial intelligence and social media algorithms in health care.

## Introduction

Digital technologies are transforming health care delivery, offering efficient, cost-effective, and low-barrier alternatives to traditional clinical services [1-3]. The ability to effectively engage with digital health services relies on digital health literacy (DHL), a critical competency that encompasses the ability to find, understand, evaluate, and apply digital health information for decision-making [4-7]. Within the broader digital transformation of society, DHL has been described as a superdeterminant of health, influencing access to health care and other social services necessary for health and well-being [2,8]. Its role has been highlighted especially when considering the rise of mis- and disinformation in informing health behaviours [9,10]. As health systems continue to integrate digital platforms for health promotion, self-management, and access to services, accurate measurement of DHL is essential for identifying gaps, informing interventions, and ensuring equitable engagement and accessibility to services across populations [2,11].

The eHealth Literacy Scale (eHEALS) is a validated 8-item measure (Cronbach α=0.88) widely used to measure DHL [4,12-14]. Initially developed as a unidimensional scale reported as a single score representative of DHL, eHEALS has been applied in numerous studies to assess individuals’ self-perceived ability to navigate online health resources [4,12,15]. However, recent psychometric analyses challenge the assumption of unidimensionality, suggesting that DHL is a multidimensional and complex construct requiring a more nuanced assessment [11,14,16-18]. This presumed unidimensionality may obscure meaningful variation in DHLs across populations, impeding capacity for targeted interventions [19,20]. Sudbury-Riley et al [11], for instance, proposed a revised 3-factor eHEALS structure for DHL aligned with social cognitive and self-efficacy theories, differentiating between awareness of online resources, skills to access them, and confidence in evaluating and applying health information [11]. While promising, this alternative model was created using data from older adults and has not been widely validated across diverse populations, including young people with whom the original scale was created [4,20]. This necessitates further investigation into its generalizability and psychometric robustness. Additionally, prior studies examining the eHEALS have primarily relied on methods that assume continuous data, despite the ordinal nature of the eHEALS response format. Further, many researchers have called for more objective and performance-based measures of DHL, moving beyond purely self-reported instruments, to better capture real-world competencies and their influence on access to digital health services [20,21]. Yet, the widespread use of the eHEALS in national and international surveys necessitates not only reconsideration of this tool but also revision in terms of multidimensionality to harness existing data [22]. Such reviews can yield nuanced insights that can inform targeted, evidence-based interventions [20].

Given these gaps, our study aims to identify and validate a revised multidimensional structure of eHEALS using exploratory factor analysis (EFA) and confirmatory factor analysis (CFA) with polychoric ordinal estimation [23]. This approach accounts for the ordinal nature of eHEALS items, providing a more accurate and precise assessment of DHL dimensions that can be implemented in real-world practice. By improving the measurement of DHL, this secondary analysis seeks to contribute much needed knowledge to enhance our ability to identify populations requiring interventions, inform intervention planning and development, and refine public health strategies aimed at improving the use and impact of digital health services among diverse populations. Our findings will contribute to a stronger empirical foundation for DHL assessment and intervention planning, ensuring that digital health resources are accessible and usable by diverse populations.

## Methods

### Study Design and Setting

This study is a secondary analysis of data from the 2022 GetCheckedOnline community survey, a cross-sectional survey designed to assess awareness and use of the service in communities where it is available. As part of the survey, we assessed DHL using the eHEALS scale as one of the determinants of awareness and use of the service. GetCheckedOnline is a publicly funded digital sexual health service operated by the British Columbia Centre for Disease Control since 2014 [24,25]. The service is available in nine urban, suburban, and rural communities across British Columbia and provides low-barrier access to sexually transmitted and blood-borne infection (STBBI) testing [24]. It allows users to create an account online, complete a sexual risk assessment, generate a laboratory test requisition, and submit specimens at a designated laboratory, with results accessible online or via public health follow-up [25].

### Study Participants and Recruitment

Survey participants were recruited through a combination of online and in-person sampling strategies to ensure diverse representation. Online recruitment involved geo-targeted digital advertisements, local message boards, community-specific Facebook groups and local community organizations’ social media accounts, as well as QR codes placed on outreach materials in community locations. In-person recruitment used venue-based sampling at public locations and events (eg, music festivals, Pride parades, and harm reduction breakfasts). We also conducted snowball sampling facilitated through community leaders and partner agencies. Eligible participants were English-speaking residents of British Columbia aged ≥16 years who reported being sexually active (defined as oral, vaginal, or anal sex with at least one partner in the past year). Individuals who had previously completed the survey or did not reside in BC based on their forward sortation area codes were excluded.

### Data Collection Instrument

The survey was conducted between July and September 2022 and was collaboratively designed by the GetCheckedOnline team in consultation with a Community Advisory Board, which included individuals with lived experience of STBBI testing within BC and those working with community organizations serving populations most affected by STBBIs. The questionnaire collected information on DHL, sociodemographic characteristics, digital technology access and use, and experiences with sexual health and STBBI testing. DHL was assessed using the eHEALS scale. Responses were collected on a 5-point Likert scale, with higher scores indicating greater DHL.

### Data Analysis

Descriptive analyses were conducted to summarize participants’ characteristics, including age, gender, ethnicity, education, income, and digital access. The distributions of eHEALS item responses were assessed using medians and IQRs, given the ordinal nature of the data. Item-level correlations were examined using polychoric correlations, which better capture relationships between ordinal variables than Pearson correlations. A correlation matrix was visualized using a heat map to explore patterns of association between eHEALS items. EFA was conducted to determine the underlying structure of the eHEALS scale.

Sampling adequacy was assessed using the Kaiser-Meyer-Olkin (KMO) measure, with values above 0.80 indicating strong factorability. Bartlett’s test of sphericity was performed to assess whether item correlations were sufficiently large for factor analysis. Factor extraction was conducted using polychoric correlation matrices and the weighted least squares means, and variance adjusted estimator, which is appropriate for ordinal data [26]. The number of factors was determined using parallel analysis, eigenvalues, and the scree plot, and factor loadings were examined using oblique rotation to account for potential correlations between factors [27]. CFA was conducted using structural equation modeling to validate the factor structure identified in the EFA.

Multiple models were tested, including a one-factor model and three-factor models based on theoretical and empirical considerations. Model fit was evaluated using several indices, including the comparative fit index (CFI) and Tucker-Lewis index (TLI), with values above 0.95 indicating good fit [28]. The root-mean-square error of approximation (RMSEA) and its 90% CI were used to assess model parsimony, with values below 0.08 considered acceptable. The standardized root-mean-square residual (SRMR) was also examined, with values below 0.05 suggesting good fit [28]. Where there were significant cross-loadings, we examined model fit indices and selected models with better fit indices and optimal factor loadings. Choices were informed by theoretical plausibility and evidence from previous studies [11,14]. Missingness across eHEALS items ranged from 12.61% to 13.46%. Patterns and mechanisms of missingness were examined prior to analysis. The Little MCAR test was conducted to evaluate whether data were missing completely at random. The test indicated significant deviation from the MCAR assumption (*χ*²_3527_= 5702, *P*<.001), suggesting that missingness was not completely random. As missingness was therefore unlikely to be completely random, multiple imputation using chained equations was applied under a missing-at-random assumption to minimize potential bias and preserve statistical power [29-31]. Sensitivity analyses were performed to compare results from the imputed dataset with a complete-case analysis.

Reliability and validity analyses were conducted to assess internal consistency and construct validity. Polychoric ordinal α and McDonald ⍵ were calculated for each factor to assess internal consistency, with values above 0.70 indicating acceptable reliability. Construct validity was evaluated through convergent validity, assessed using average variance extracted (AVE), with values above 0.50 suggesting good convergence [32]. Discriminant validity was assessed by ensuring that AVE values exceeded the squared correlations between factors. All analyses were conducted using R (version 4.3.1), with Lavaan used for structural equation modeling, psych for EFA and reliability analyses, and Corrplot for correlation visualizations [33,34].

### Ethical Considerations

This study was reviewed and approved by the University of British Columbia Behavioral Research Ethics Board (REB#H25-01028) allowing for secondary analysis of previously collected anonymous data without additional consent. The original 2022 community survey data obtained informed consent from all participants. Survey participants were offered the option to enter a draw for one of five CAD $100 (US $71) Visa gift cards by providing separate contact information that was not linked to their survey responses. The dataset used for this analysis was fully deidentified before access, and no personal identifiers were available to the research team. No identifiable images or other personal data are included in this manuscript or its supplementary materials. The study adhered to STROBE (Strengthening the Reporting of Observational Studies in Epidemiology) checklist for cross-sectional studies (Multimedia Appendix 1), ensuring transparency and rigor in reporting [35].

## Results

### Description of Participants

Among 2720 initial survey respondents, 1657 met the inclusion criteria, with a mean age of 33.0 (SD 11.77) years. Among these, 47.3% (n=784) identified as women, 14.5% (n=240) as gender identities characterized as gender diverse, 30.4% (n=503) as belonging to a racialized minority group, 49.5% (n=820) as a sexual minority, and 39.4% (n=653) as straight. Regarding education, 29.9% (n=495) had a postsecondary education and 32.2% (n=534) had a bachelor’s degree or higher. Among respondents 23.5% (n=389) earned <CAD $20,000 per annum while 80.5% (n=1334) reported easy access to the internet (Table 1).

**Table 1:** Sociodemographic and digital access characteristics of respondents (N=1657) to the 2022 GetCheckedOnline Community Survey (British Columbia, Canada).

Characteristic			Values^a^
Age (years), mean (SD); 95% CI			33.00 (11.77); 32.4-33.6
**Gender, n (%); 95% CI**			
	Man	470 (28.4); 26.2-30.6	
	Woman	784 (47.3); 44.9-49.7	
	Gender-diverse^b^	240 (14.5); 12.9-16.3	
	Not disclosed	163 (9.8); 8.5-11.4	
**Ethnicity, n (%); 95% CI**			
	Racialized minority	503 (30.4); 28.1-32.6	
	White	892 (53.8); 51.4-56.2	
	Not disclosed	262 (15.8); N/A^c^	
**Sexuality, n (%); 95% CI**			
	Nonheterosexual^d^	820 (49.5); 47.1-51.9	
	Heterosexual	653 (39.4); 37.1-41.2	
	Not disclosed	184 (11.1); 9.7-12.7	
**Education, n (%); 95% CI**			
	High school or less	415 (25.0); 23.0-27.2	
	Postsecondary education	495 (29.9); 27.7-32.1	
	Bachelor’s degree or higher	534 (32.2); 30.0-34.5	
	Not disclosed	213 (12.9); 11.3-14.6	
**Income per annum (CAD $; CAD $1=US $0.71); n (%); 95% CI**			
	<20,000	389 (23.5); 21.5-25.6	
	20,000-39,000	318 (19.2); 17.4-21.2	
	40,000-59,000	284 (17.1); 15.4-19.0	
	60,000-79,000	178 (10.7); 9.3-12.3	
	80,000 or more	175 (10.6); 9.2-12.1	
	Not disclosed	313 (18.9); 17.1-20.8	
**Ease of going online (digital access), n (%); 95% CI**			
	Easy	1334 (80.5); 78.5-82.3	
	Not easy	131 (7.1); 6.7-9.3	
	Not disclosed	192 (11.6); 10.1-13.2	
**Survey recruitment channel, n (%); 95% CI**			
	In-person	1057 (63.8); 61.4-66.1	
	Online	600 (36.2); 33.9-38.6	

^a^Values are presented as mean (SD) with 95% CIs for continuous variables and n (%) with 95% binomial CIs for categorical variables. CIs for means were calculated using the t-distribution; proportions were estimated using the Wilson method.

^b^Includes gender-fluid, gender queer, agender, and nonbinary participants who selected a combination of more than one gender categories.

^c^N/A: not applicable.

^d^Includes asexual, bisexual, gay, lesbian, heteroflexible, pansexual, queer, and others.

### eHEALS Item Analysis

**Figure 1:**
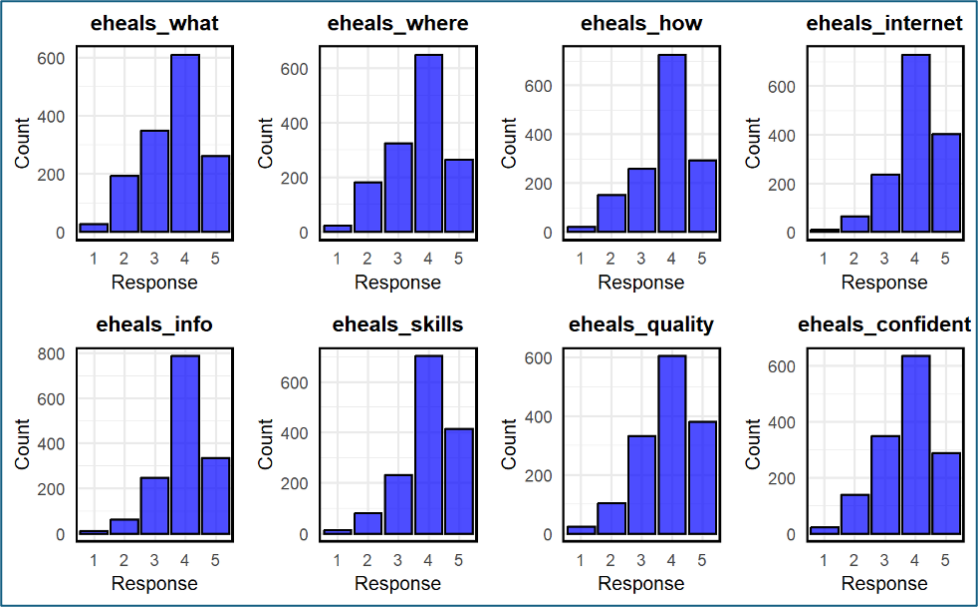
Distribution of responses to the eHEALS items. eHEALS: eHealth Literacy Scale.

**Figure 2:**
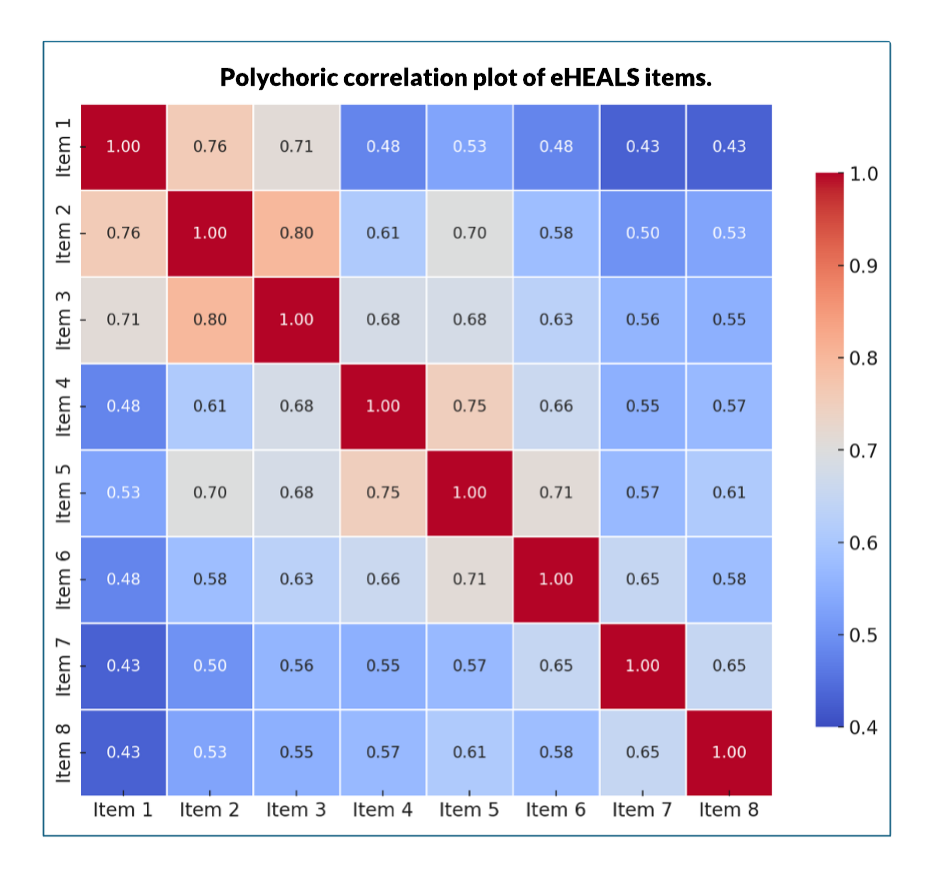
Polychoric correlation plot of the eHEALS items. eHEALS: eHealth Literacy Scale.

The mean scores for the eight eHEALS items ranged from 3.62 (SD 0.99) to 4.01 (SD 0.83) and a median score of 4.0 out of a possible score of 5, indicating most respondents had moderate to high agreement with digital health literacy statements (Table 2 and Figure 1). The internal consistency of the scale was excellent, with a polychoric ordinal α of 0.92 and an average inter-item correlation of 0.60, supporting the reliability of the measure for ordinal data (Figure 2).

**Table 2:** Distribution of responses to eHEALS (eHealth Literacy Scale) items assessing digital health literacy among respondents to the 2022 GetCheckedOnline Community Survey (polychoric ordinal a=0.92; average correlation=0.60).

eHEALS item	Description	Score, mean (SD)	95% CI	Score, median (IQR)^a^
Item 1: eHEALS_what	I know what health resources are available on the Internet	3.62 (0.99)	3.56-3.67	4.0 (1.0)
Item 2: eHEALS_where	I know where to find helpful health resources on the Internet	3.66 (0.97)	3.61-3.71	4.0 (1.0)
Item 3: eHEALS_how	I know how to find helpful health resources on the Internet	3.77 (0.93)	3.72-3.82	4.0 (1.0)
Item 4: eHEALS_internet	I know how to use the Internet to answer my questions about health	4.01 (0.83)	3.97-4.05	4.0 (1.0)
Item 5: eHEALS_info	I know how to use the health information I find on the Internet to help me	3.95 (0.80)	3.91-3.99	4.0 (0.0)
Item 6: eHEALS_skills	I have the skills I need to evaluate the health resources I find on the Internet	3.99 (0.87)	3.94-4.03	4.0 (1.0)
Item 7: eHEALS_quality	I can tell high quality health resources from low quality health resources on the Internet	3.84 (0.95)	3.79-3.89	4.0 (2.0)
Item 8: eHEALS_condifident	I feel confident in using information from the Internet to make health decisions	3.72 (0.94)	3.67-3.77	4.0 (1.0)

^a^Medians and IQRs are reported for skewed variables without CIs.

### Factor Analysis

**Figure 3:**
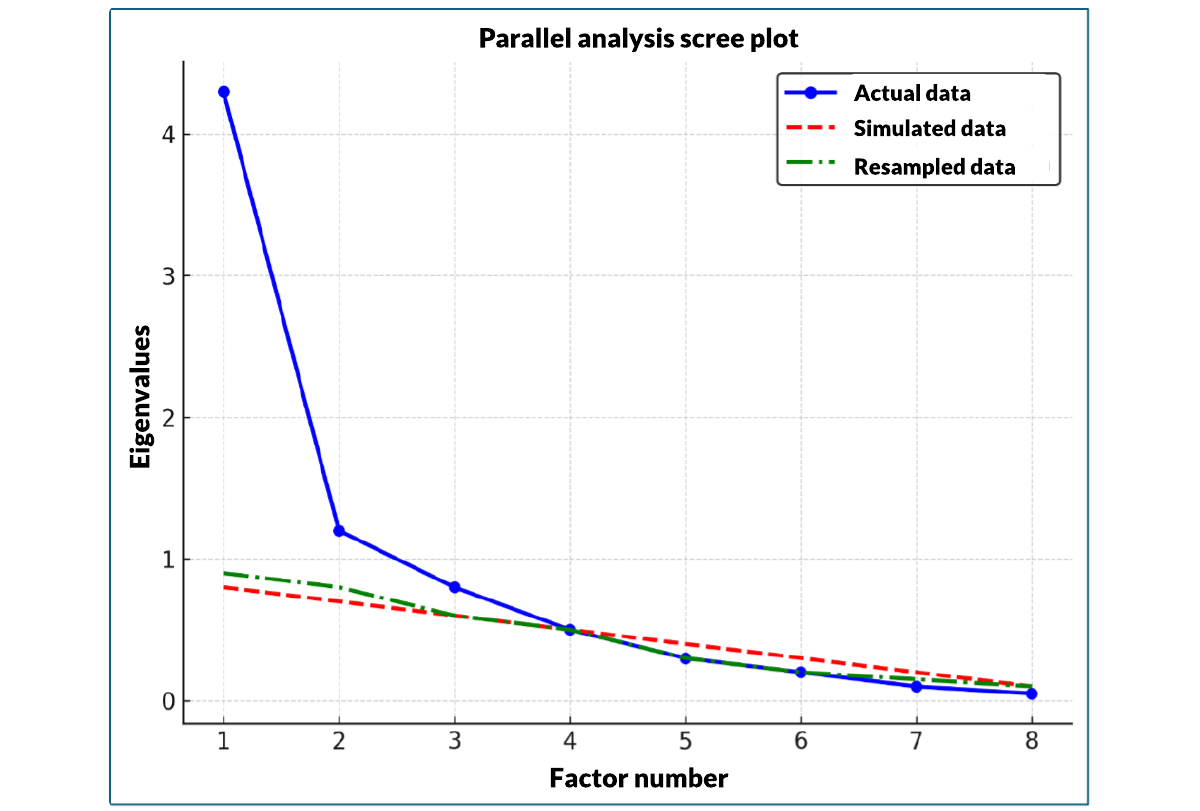
Parallel analysis scree plot for eHEALS items.

The KMO test demonstrated an overall sampling adequacy for factor analysis of 0.89 with values for individual items ranging from 0.86 to 0.91. The Bartlett test of sphericity revealed significant findings (*χ*²_28_=7222.8, *P*<.001), supporting the suitability of factor analysis. EFA supported a three-factor solution with eigenvalue of >1 as demonstrated by a parallel analysis scree plot (Figure 3), explaining 65.7% of the variance within the dataset. We also noted potential cross-loading with item 8 loading across 2 out of the 3 factors (Table 3).

**Table 3:** Standardized factor loadings for the three-factor eHEALS (eHealth Literacy Scale) model based on exploratory factor analysis of responses to the 2022 GetCheckedOnline Community Survey.

Item	Information navigation	Resource appraisal	Confidence in use	Communalities (h²)
Item 1: eHEALS What	0.91	—^a^	—	0.69
Item 2: eHEALS Where	0.85	—	—	0.85
Item 3: eHEALS How	0.66	—	—	0.78
Item 4: eHEALS Internet	—	0.83	—	0.71
Item 5: eHEALS Info	—	0.88	—	0.81
Item 6: eHEALS Skills	—	0.64	—	0.66
Item 7: eHEALS Quality	—	—	0.99	0.99
Item 8: eHEALS Confident	0.42	—	0.35	0.54

^a^Not applicable.

In CFA, the 1-factor model (originally proposed by Norman and Skinner [4]) exhibited a poor fit to the data (*χ*²_20_=849.208, *P*<.001, RMSEA=0.158, SRMR=0.072, CFI=0.987, TLI=0.981; Table 4). The three-factor model proposed by Sudbury-Riley et al [11] revealed improved fit to the data (*χ*²_17_=420.215, *P*<.001, RMSEA=0.127, SRMR=0.041, CFI=0.935, TLI=0.894). However, the EFA-informed structure (model 3), assuming continuous variables, resulted in a substantial improvement in fit indices (*χ*²_16_=163.558, *P*<.001, RMSEA=0.077, SRMR=0.025, CFI=0.977, TLI=0.961). The final model (model 4), incorporating the same EFA-informed three-factor structure but accounting for the ordinal nature of eHEALS responses, exhibited the best model fit (*χ*²_17_=71.680, *P*<.001, RMSEA=0.059, SRMR=0.026, CFI=0.969, TLI=0.948), supporting the appropriateness of a three-factor solution while treating eHEALS items as ordinal data.

**Table 4:** Model fit indices for competing factor structures of the eHEALS (eHealth Literacy Scale) based on confirmatory factor analysis of responses to the 2022 GetCheckedOnline Community Survey.

Model	Responses, n	Chi-square (*df*)	*P* value	RMSEA^a^	90% CI	SRMR^b^	CFI^c^	TLI^d^
Model 1: 1-Factor model (multiple imputation)	1657	849.208 (20)	<.001	0.158	0.149-0.167	0.072	0.987	0.981
Model 2: 3-Factor model (model of Sudbury-Riley et al [11] assuming continuous variables)	1461	420.215 (17)	<.001	0.127	0.117-0.138	0.041	0.935	0.894
Model 3: 3-Factor model (identified from EFA^e^, assuming continuous variables)	1461	163.558 (16)	<.001	0.077	0.066-0.088	0.025	0.976	0.959
Model 4: 3-Factor model (identified from EFA, assuming ordinal variables with multiple imputation)	1657	71.680 (17)	<.001	0.059	0.049-0.070	0.026	0.969	0.948

^a^RMSEA: root-mean-square error of approximation.

^b^SRMR: standardized root-mean-square residual.

^c^CFI: comparative fit index.

^d^TLI: Tucker-Lewis index.

^e^EFA: exploratory factor analysis.

### Factor Structure and Standardized Loadings

The final three-factor model of the eHEALS scale consisted of the following domains, which we describe as Information Navigation, Resource Appraisal, and Confidence in Use. The standardized factor loadings from CFA are summarized in Table 5. Information Navigation reflects participants’ ability to locate and access online health resources effectively with factor loadings for this domain ranging from 0.765 to 0.917 (ie, strong relationships between items and the underlying construct). Resource Appraisal assesses respondents’ capacity to evaluate the reliability and relevance of online health information. Items within this factor demonstrated factor loadings ranging from 0.825 to 0.892, reflecting good construct validity. Confidence in use represents the ability to apply digital health information confidently in decision-making, with factor loadings of 0.803 for both corresponding items. The standardized loadings suggested that all items load significantly onto their respective latent factors, demonstrating the validity of the three-factor structure. Additionally, all factor loadings exceeded 0.70, which is considered strong evidence for convergent validity.

**Table 5:** Standardized factor loadings for the three-factor eHEALS (eHealth Literacy Scale) model based on confirmatory factor analysis of responses to the 2022 GetCheckedOnline Community Survey.

Factor	eHEALS item	Standardized factor loading
Information Navigation	Item 1: eHEALS What	0.765
Information Navigation	Item 2: eHEALS Where	0.909
Information Navigation	Item 3: eHEALS How	0.917
Resource Appraisal	Item 4: eHEALS Internet	0.839
Resource Appraisal	Item 5: eHEALS Info	0.892
Resource Appraisal	Item 6: eHEALS Skills	0.825
Confidence in Use	Item 7: eHEALS Quality	0.803
Confidence in Use	Item 8: eHEALS Confident	0.803

### Construct Reliability and Average Variance Explained

Table 6 summarizes the internal consistency of the three factors using composite reliability and AVE. The composite reliability values ranged from 0.784 to 0.900, exceeding the recommended threshold of 0.70, suggesting adequate reliability. The AVE values assessing the proportion of variance captured by each latent factor relative to measurement error, ranged from 0.503 to 0.738, supporting good convergent validity.

**Table 6:** Construct validity and reliability indices for the three-factor eHEALS (eHealth Literacy Scale) model based on responses to the 2022 GetCheckedOnline Community Survey.

Factor	Average variance explained	Composite reliability
Information Navigation	0.661	0.900
Resource Appraisal	0.738	0.888
Confidence in Use	0.503	0.784

## Discussion

### Principal Findings

In this study, we identified and validated a multidimensional structure of the eHEALS, confirming that based on the existing eHEALS questionnaire, DHL is best conceptualized through three distinct but related factors: Information Navigation, Resource Appraisal, and Confidence in Use. Information Navigation describes an individual’s ability to locate and retrieve health-related information online, Resource Appraisal reflects their capacity to critically assess the quality and credibility of digital health content, and Confidence in Use captures their self-assurance in using online information for health decision-making. The final three-factor model demonstrated excellent fit, construct validity, and reliability reinforcing the utility of this revised eHEALS in assessing DHL. The strong fit indices for the three-factor model—particularly when accounting for the ordinal nature of the eHEALS questionnaire—highlight the importance of appropriately modeling the categorical nature of DHL survey items. The validation of this refined factor structure offers a more precise measurement framework for assessing DHL across diverse populations.

### Comparison With Previous Studies and Literature

Previous research has largely treated eHEALS as a unidimensional scale, measuring digital health literacy as a singular construct [4,12]. However, emerging evidence suggests that DHL is multifaceted, requiring distinct skills for accessing, understanding, evaluating, and applying health information in online contexts [5,11,16,36,37]. Our results align with and expand upon prior studies that have questioned the original unidimensional structure of eHEALS, supporting the need for a revised framework that captures different facets of DHL [11,36].

Sudbury-Riley et al [11] proposed an alternate three-factor structure for eHEALS based on socio-cognitive and self-efficacy theory to suggest DHL consists of awareness, skills, and evaluation capabilities [11]. Others have suggested two- or three-factor structures for eHEALS [16,17,38]. Our model advances multidimensional conceptualizations of the eHEALS by explicitly accounting for the ordinal nature of the scale, particularly in the context of widespread digital transformation, which has contributed to nonnormal response distributions. The latent factors identified in this analysis reflect key components of Bandura’s Social Cognitive Theory, which emphasizes the role of personal capabilities, environmental factors, and social influences in shaping knowledge acquisition [5,39]. Our results also align with a recent conceptual model of DHL, which identified four main attributes including goal-driven regulation, information processing, usage, and communication [37]. Our findings indicate that information navigation, resource appraisal, and confidence in use are distinct yet interrelated constructs, supporting the view that DHL is not simply about information retrieval but extending to include the ability to critically engage with and apply online health information [5,37]. Interventions to promote DHL need to not only focus on the peoples’ self-efficacy and personal capabilities through tutorials and demonstrations, but also community-level and social factors that can promote DHL. For example, at the interpersonal level, there is emergent evidence of the impact of peer support networks in enhancing marginalized communities’ collective trust in digital health technology which ultimately influences DHL [40,41]. At the community-level, public goods like library services have been leveraged to promote training and vocational skills that can contribute to DHL [42]. Beyond these efforts must address upstream factors including digital access and online regulatory policies that influence community trust [40,41].

### Novelty of Findings and Contributions

This study validates the multidimensional factor structure of eHEALS using advanced psychometric techniques and multiple imputation within a general population as compared to others that have not recognized the ordinal nature of the survey and have been conducted in subsets of the population (eg, among older adults) limiting their generalizability [11,17]. By leveraging EFA ordinal CFA and polychoric correlations, we provide strong empirical evidence for a revised three-factor structure of the eHEALS scale that accounts for the ordinal structure of the data. This revised structure provides greater conceptual clarity and measurement precision, making it particularly useful for researchers, clinicians, and public health practitioners seeking to assess DHL.

### Public Health Implications

Within the context of societal digital transformation, there has been increasing recognition of the need for improved measures of DHL to guide public health advocacy, policy, and action at multiple levels [2,8,43,44]. DHL is now considered a fundamental determinant of health, shaping individuals’ ability to leverage health information in an increasingly digital world [8,40,41]. At a population level, a more nuanced understanding of DHL can inform better-targeted interventions by identifying specific gaps in information-seeking skills, critical evaluation of online content, or confidence in applying health information as described above. Traditionally, digital literacy interventions have focused on improving access and navigation; yet, emerging evidence suggests that developing appraisal skills and trust in credible sources is equally critical. These skills have become particularly important in light of the proliferation of online mis- and disinformation, which has significantly increased since the COVID-19 pandemic, fueling vaccine hesitancy and the re-emergence of vaccine-preventable diseases such as measles [9,45].

At a program level, this validated three-factor structure of DHL can serve as a planning and evaluation tool for digital health programs, enabling more precise assessment of intervention effectiveness and guiding ongoing program adaptations [46]. This is particularly relevant for public health initiatives targeting equity-deserving populations, who may experience unique barriers to engaging with digital health tools [46,47]. For instance, interventions aimed at populations with poorer resource appraisal skills may need to incorporate structured training on misinformation detection, fact-checking strategies, and trust-building in digital health sources [10]. To maximize their impact, adapted DHL measures must be considered in national surveys like the Canadian Community Health Survey [48].

### Future Research Directions

Future research should examine the generalizability of this three-factor model across diverse populations, including different age groups, cultural and linguistic contexts, and health conditions. Longitudinal studies are particularly needed to assess how DHL evolves over time, its impact on real-world health behaviors, and its role in mediating or moderating sociodemographic disparities in access to and use of digital health interventions. Furthermore, as with increasingly digital-first health services and with increasing integration of artificial intelligence and algorithm-driven health information, refining DHL measures will be essential [10,49,50]. This revised scale serves as a foundation for developing more precise and contemporary assessments that capture the complexities of DHL in an era of rapidly advancing health technologies.

### Strengths and Limitations

This study has several notable strengths. First, we conducted a rigorous psychometric evaluation, incorporating both exploratory and confirmatory factor analysis, to validate the revised eHEALS structure. Our approach leveraged polychoric ordinal factor analysis, which appropriately models the categorical nature of eHEALS items, ensuring more accurate measurement. The large and diverse sample enhances the generalizability of our findings across different populations. Recruiting over 60% of participants via in-person channels contributed to ensuring a diverse sample.

However, some limitations should be acknowledged. Our study relied on self-reported measures of DHL, which may be subject to response bias. This is a widely debated issue, given evidence suggesting that survey respondents may overestimate their DHL [51,52]. The original survey was limited to English-speaking participants and relied on a convenience sample of individuals who were willing to participate, introducing potential self-selection bias and limiting the generalizability of the findings. Therefore, although the three-factor model demonstrated excellent model fit, further validation across diverse populations, including non–English-speaking groups and different health care contexts, is needed to ensure generalizability. This is especially relevant as our survey targeted populations accessing testing for sexually transmitted infections with estimated DHLs being significantly higher than the Canadian average [22]. While most fit indices were excellent, slightly lower construct reliability for the factor Confidence in Use suggests the need for more reliable scales that are dependent on more refined items. Moreover, given the rapid evolution of digital technologies, findings reinforce the need for continued refinement of DHL measures to better capture emerging competencies required for navigating modern digital health ecosystems.

### Conclusion

This study validates a revised three-factor structure of the eHEALS, confirming that DHL comprises three distinct but interrelated competencies: Information Navigation, Resource Appraisal, and Confidence in Use. These findings enhance conceptual clarity in measuring DHL, providing stronger links to the DHL literature and an empirical foundation for future research and intervention development. As digital health platforms continue to play an increasingly central role in health care and public health, the ability to accurately assess and target DHL is essential for promoting informed health decision-making. The validated three-factor model offers a more precise tool for identifying specific gaps in DHL, enabling tailored interventions that equip individuals with the skills needed to navigate, evaluate, and apply digital health information effectively. However, further research is required to explore how these factors influence access to digital health services, while ongoing efforts must focus on developing updated scales that account for emerging technological advancements, including the growing influence of artificial intelligence and social media in health information ecosystems.

## Appendix

Multimedia Appendix 1 STROBE checklist for cross-sectional studies.

## Abbreviations

AVE: average variance extracted
CFA: confirmatory factor analysis
CFI: comparative fit index
DHL: digital health literacy
EFA: exploratory factor analysis
eHEALS: eHealth Literacy Scale
KMO: Kaiser-Meyer-Olkin
RMSEA: root-mean-square error of approximation
SRMR: standardized root-mean-square residual
STBBI: sexually transmitted and blood-borne infections
STROBE: Strengthening the Reporting of Observational Studies in Epidemiology
TLI: Tucker-Lewis index

## Data Availability

The datasets analyzed in this study are part of organizational improvement efforts and are not publicly available. They may be accessed from the corresponding author upon reasonable request.
